# Evaluation of biological activity via biospeckle laser imaging

**DOI:** 10.52601/bpr.2025.250010

**Published:** 2026-02-28

**Authors:** Mohammad Zaheer Ansari

**Affiliations:** 1 School of Basic and Applied Sciences, Department of Physics, Raffles University, Neemrana, Rajasthan 301705, India

**Keywords:** Biospeckle, Laser speckle imaging, Tissue, Microcirculation, Activity

## Abstract

We describe the statistical characteristics of optical speckle patterns formed by illuminating biological tissues, commonly called biospeckles. The predominant techniques used to gather information from the movement of speckle patterns are detailed. Using vegetable tissues, we monitored the senescence process and created vascularization maps of leaf tissues. The Fujii method, which has been modified, has emerged as the most effective approach for highlighting the biological activity across leaf tissues. This technique relies on the presence of fluid flow to create highly detailed maps of tissue microcirculation. The method of temporal contrast evaluation produced a significant spectral activity map, which allowed for the detection of both instant and invisible bruised tissue. The evaluation revealed that biological specimens can exhibit a unique time history of speckle pattern (THSP) patterns, which may serve as a biological signature for the sample. Additionally, an activity index was calculated to define the assay’s activity under different biological conditions, and the results were tested and verified across multiple samples.

## INTRODUCTION

When a coherent light beam is scattered by or transmitted through a diffuse surface, the formation of a granular structure occurs in the free space. We refer to this phenomenon as speckles. The resulting pattern evolves in time if the scatterer surface generates changes in the optical path of the interfering rays, such as micrometric motion or tiny alterations in the refraction index. This temporal modulation provided information on the activity of the sample. Dynamic speckle, an interference pattern produced by the movement of molecules and microscopic structures (scatterers) as a result of this activity, can be used to detect minute changes in the biological activity of cells and tissues (Braga *et al*. 2009).

This condition occurs when speckle patterns are dispersed from recently painted surfaces, during rusting processes, in liquids containing particles moving in Brownian motion, or from living tissues, where it is possible to observe the microflow of biological liquids and the motion of cells (da Silva and Muramatsu 2008).

**Figure 1:**
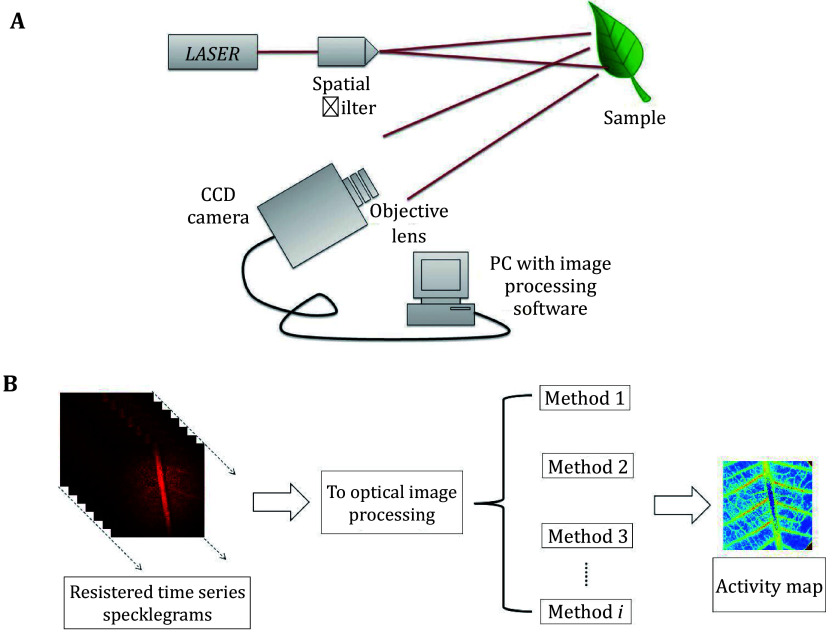
**A** Biospeckle imaging configuration for qualitative evaluation of the biological tissue. **B** A block diagram showing acquisition of time series speckle frames, showing generation of activity map

When coherent light from a live body is scattered and forms a pattern of dynamic interference on the detector, it is called a laser biospeckle (Aizu *et al*. 1996). Plants emit two types of light: one type comes from deep light within the leaves and backscattered light from the surface. As Fig. 1 illustrates, speckles were produced by both types of dispersed light. They are known as biospeckles because the movement of internal organelles and cellular processes like the transport of water and nutrients cause these speckles to fluctuate in intensity at random. The quality and maturity of fruits and vegetables, seed viability analysis, internal fruit loss detection, and other aspects of plant activity can be monitored by looking at the dynamic changes of the biospeckle (Braga 2017; Minz and Nirala 2014; Zdunek *et al*. 2014). It has also been used in agriculture.

Agriculture, medicine, and industrial operations are just a few scientific fields that have used the dynamic laser speckle approach. White *et al*. calculated the functional vascular density (White *et al*. 2011), Mavilio *et al*. researched the paint drying process (Mavilio *et al*. 2010), and Ansari and Nirala are among the recent uses of this technology (Ansari and Nirala 2014). Several image-processing and signal-processing techniques have also been used to analyze optical interference patterns.

Many graphical and numerical methods can be employed to analyze data from optical interference patterns (Rabal and Braga 2018). The type of signals collected dictates which particular analysis method should be used for biospeckle data. For homogeneous matter, the most effective approaches are numerical ones based on first- and second-order statistics, such as inertia moment (IM) (Arizaga *et al*. 2019) or Barier's contrast (Briers 1975). However, image-processing methods such as Fujii's method (Fujii *et al*. 1987), the generalized difference (GD) approach (Arizaga *et al*. 2002), and laser speckle contrast analysis (LASCA) (Briers and Webster 1996) have been shown to be more effective in determining the type of signals released from samples when applied to non-homogeneous biological tissues.

This work provides the statistical characteristics of biospeckle, or dynamic speckles, which are optical grainy patterns created by shining light on biological tissues. The study presents the primary methods for extracting information from speckle patterns, with particular emphasis on the Fujii method, generalized difference, and temporal correlation of contrast analysis (tLASCA). The senescence process and vascularization maps on leaves are monitored by the investigation of vegetable tissues. To highlight the biological activity in all leaf tissues, the weighted parameterized Fujii (WPF) method turned out to be the most effective strategy.

The method of temporal contrast evaluation produced a significant spectral activity map, which allowed for the detection of both instant and invisible bruised tissue. A qualitative evaluation of the time-history speckle pattern (THSP), followed by a co-occurrence matrix (COM) calculation, indicated that biological specimens can possess their own unique THSP patterns, which could serve as a biological signature for the sample. Finally, an activity index defining the essay’s activity under different biological conditions was calculated and the results were tested and verified for several samples.

The study explores the use of biospeckle laser imaging to evaluate biological activity in plant tissues and fruits. It presents multiple case studies — mapping microcirculation in leaves, monitoring fruit aging, detecting bruises, and assessing tissue oxidation — to demonstrate the versatility of the technique. We introduce a WPF algorithm that appears to improve the visualization of speckle activity in leaf veins. The inclusion of various analysis methods and comparative images strengthens the study, providing a comprehensive perspective on biospeckle data processing. The results are consistent with expectations, supporting the validity of the approach.

## RESULTS AND DISCUSSION

We present the results of various biospeckle experiments on several different biological specimens.

### Microcirculation mapping in vegetal leaves

**Figure 2:**
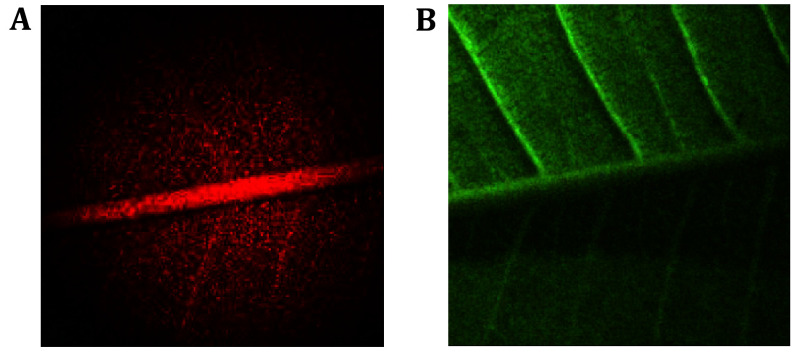
A typical frame from biospeckle sequence (**A**), with the original object (*Plumeria* tree leaf) (**B**)

Micro circulation map was generated using the classic data collection of *Plumeria rubra* leaf images (Ansari and Nirala 2015). For the experiment, a backward scattering configuration was used to illuminate the five surfaces of the *Plumeria rubra* leaves. Immediately after removal, fresh, healthy green *Plumeria* (common name *Frangipani*) leaves were sacrificed for laser imaging. The large leaves measured approximately 12 cm in length and 6 cm in width. The leaves possessed a distinctive oblong form with a rounded apex. There were noticeable parallel secondary veins that extended from the mid-vein to the leaf margins, giving the otherwise dark, leathery leaves a shining upper surface (Fig. 2). Five fresh, healthy green *Plumeria* leaves were collected and immediately subjected to laser imaging and similar results were obtained for each sample.

After cropping, leaves exhibit varying levels of activity at different stages. Evaporation, exchanges across cell membranes, attacks by microorganisms, microflow, and other phenomena combine to form the overall activity. These processes appear tangled and complex. Experiments performed in the Fraunhoffer configuration can be used to assess using a biospeckle.

By tracking the biospeckle in the picture plane, the spatial distribution of the activity can be determined. A temporal sequence of blurry images taken from the sample under laser light illumination was the focus of the experiment. Next, using the frame differences, a new image was created by subtracting the previous image from the other. Greater disparities were seen in areas of higher activity, and the resulting graphic shows more active zones in these areas. The sequence of registers from a freshly cropped leaf is shown in the figure below (Fig. 2).

**Figure 3:**
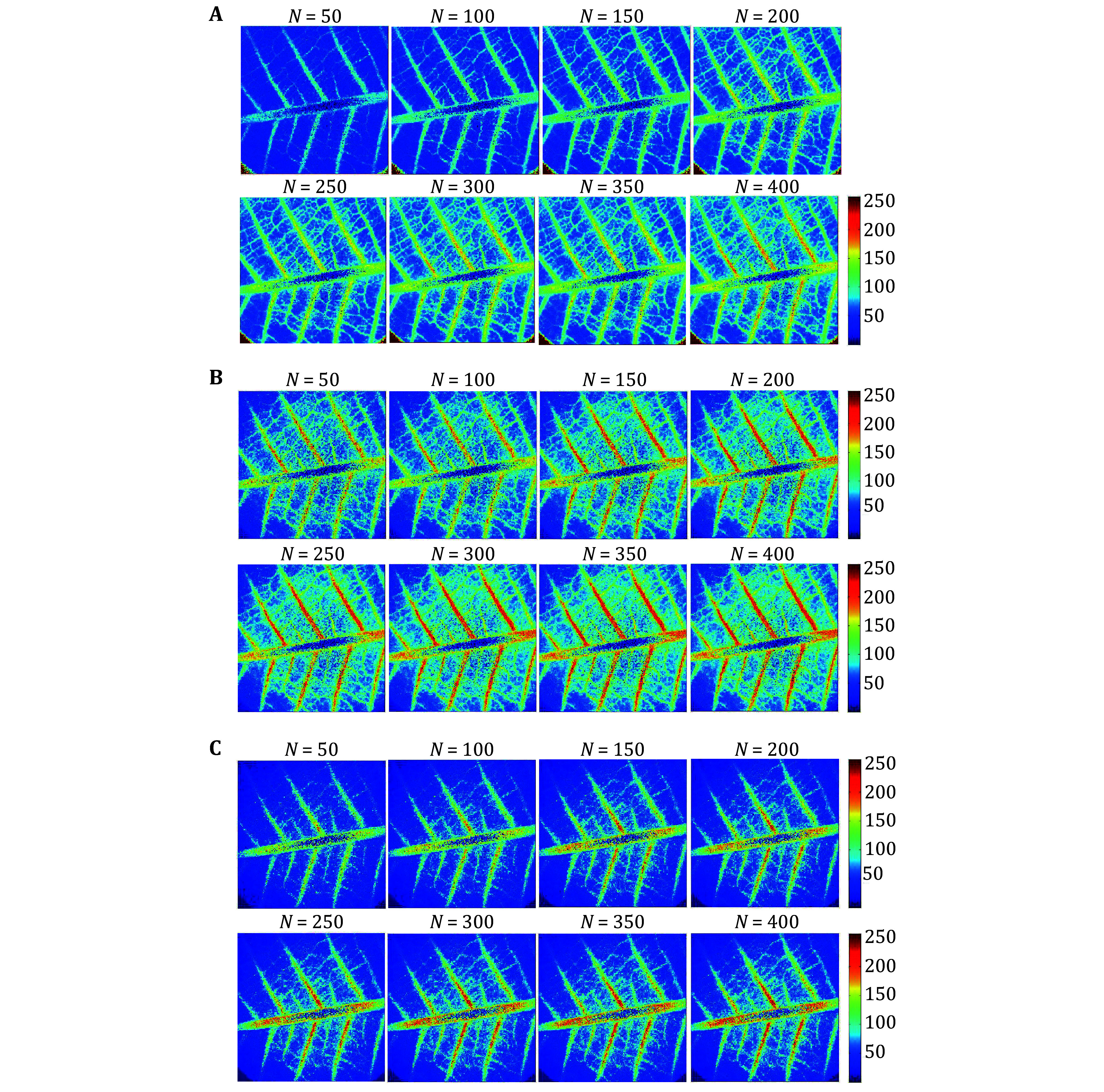
*Plumeria rubra* leaf under dynamic laser processed with weighted parameterized Fujii (with reference gray level *g*_r_ is 150) (**A**), Parameterized Fujii (with reference gray level *g*_r_ is −1) (**B**) and temporal difference (**C**). In all the images, *N* is the number of registered speckle frames

Figure 3 shows the speckle spectral maps of the leaf surface illuminated with coherent light. In contrast, as shown in Fig. 3A, they are similar to that of the temporal difference (TD) (Fig. 3C) and parameterized Fujii pictures (Fig. 3B). A generalized equation that can valorize the locations surrounding any gray level of interest should be the optimal approach. We provide a different approach to the parameterized Fujii method called WPF, which is a parameterized version of the Fujii algorithm. This technique allows the user to adjust its parameters based on the objects in the pictures that they find interesting.

The activity images shown in Fig. 3 were generated for several stack sizes (*N*). Using the WPF algorithm, we can observe the effect of the stack size on the generated activity images (Fig. 3A). While increasing the stack size (*N*), the activity related to the secondary veins was clearly visible, and the microcirculation density over the leaf tissue was well identified. However, no such improvement in the visibility of vein activity was observed with the other two routine algorithms for parameterized Fujii and TD (Figs. 3B and 3C).

Most of the activities linked to the secondary veins were hidden by the parameterized Fujii and TD methods. The WPF algorithm has an advantage over parameterized Fujii and TD in that it provides better activity maps with the proper selection of the stack size (*N*). In the current study, the stack size containing 400-time-sequenced speckle frames provided an optimum activity map of leaf tissue.

A backward scattering configuration was used to examine *Plumeria rubra* leaves. The WPF algorithm provided the most detailed activity maps, outperforming conventional Fujii and TD methods in vein visualization.

While using the WPF method, the optimal value of the reference gray level *g_r_*, should be chosen to retain the image quality in terms of resolution and contrast. Recently, we have studied in detail about how to determine the reference gray level *g_r_*, and how this parameter affects the results (Ansari and Nirala 2015).

The WPF method offers several advantages over the parameterized Fujii algorithm, particularly in biospeckle laser imaging and dynamic speckle pattern analysis. The key advantages are enhanced sensitivity, improved noise reduction, higher spatial resolution, robustness to variability, and more accurate quantification, *etc*.

The WPF method assigns weights to different regions of the speckle pattern based on their relative significance, improving the detection of subtle biological activity variations. By incorporating weighted parameters, the WPF method effectively minimizes background noise and enhances the signal-to-noise ratio, leading to clearer and more reliable results. The weighting mechanism refines the spatial representation of dynamic speckle activity, enabling more precise mapping of biological processes. The WPF method can be fine-tuned to emphasize specific activity regions, making it more adaptable for various applications, such as tissue viability assessment or microcirculation analysis. Moreover, the weighted approach reduces the impact of fluctuations in speckle patterns due to external factors like illumination variations or sample heterogeneity. By incorporating weight factors, the WPF method provides a more accurate measure of speckle dynamics, improving the reliability of quantitative biospeckle assessments.

These advantages make the WPF method a more refined and effective approach compared to the standard PF method, particularly in applications requiring high precision and sensitivity in speckle pattern analysis.

### Biospeckle activity in apple surface aging

Freshly cut apple samples were monitored over ten days using the TD method. A significant decline in activity was observed, confirming biospeckle imaging as a viable tool for monitoring fruit deterioration. Data was evaluated and tested on ten different apple samples and a similar result was obtained.

**Figure 4:**
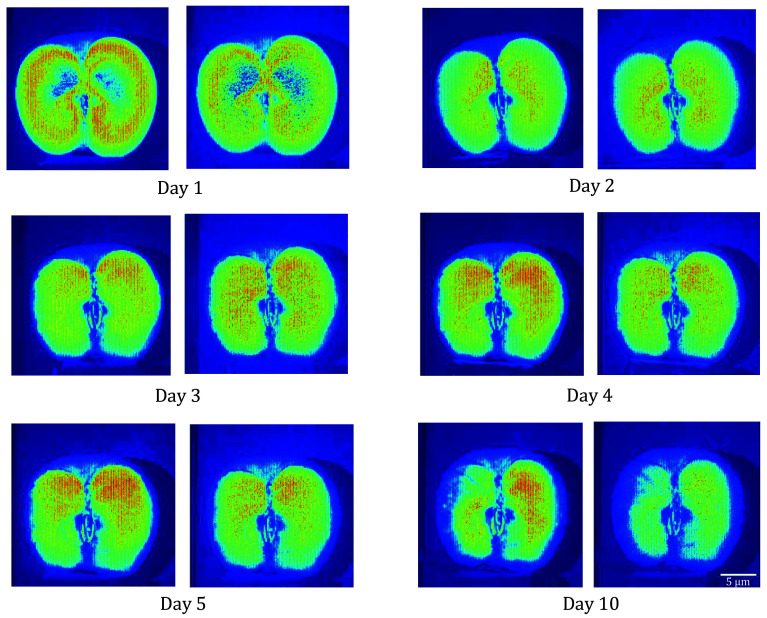
Activity images computed with the TD method under dynamic laser with exposure time, *t* = 1 s (left panel), and *t* = 100 ms (right panel)

Figure 3 displays the time evolution of the biological activity of a fresh-cut apple over a shelf-life period of ten days of storage at room temperature (25°C). During the first day, the sample shows a higher activity. The corresponding activity images were generated using a TD method as described in Eq. 2 (shown in the Material and Methods section). One can observe a degradation in the activity of the sample during the ten days. The color bar indicates the activity levels with blue for the low activity and red for the high activity. The activity was evaluated at two different exposure times of the camera. The left panel of Fig. 4 shows images generated at an exposure time of 1 s while on the right panel, one can see the images at 100 ms. The selection of two different exposure times doesn’t make many differences between activity images of the assay and thus a minimal exposure of 1 s (of the recording) can be chosen when evaluating the activity of fresh-cut fruits using biospeckle imaging.

**Figure 5:**
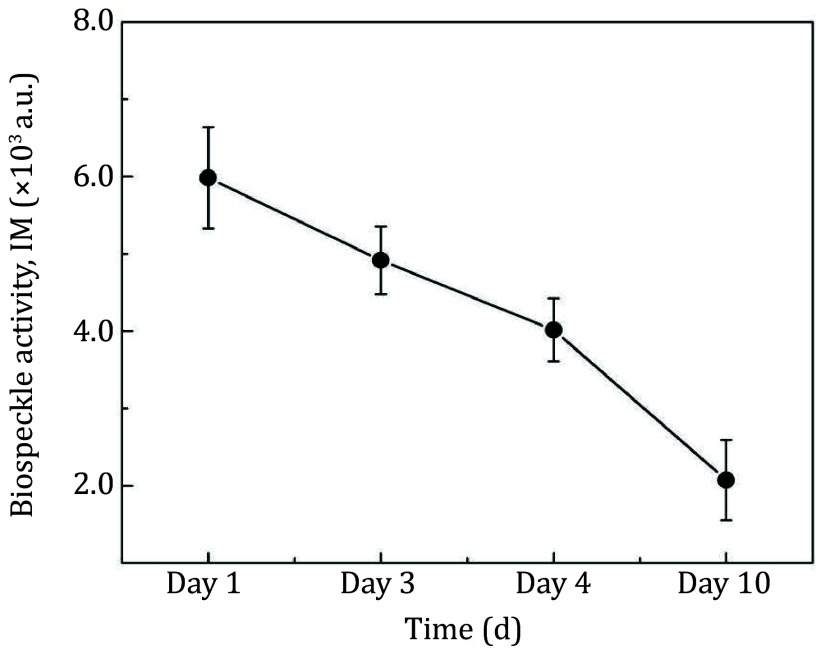
Evolution of biospeckle activity over ten days of shelf-life storage of cut apple. The error bar indicates SE

Figure 5 presents a quantitative evaluation of the activity of the same cut apple over ten days of shelf-life storage. It shows a degradation of the activity of the assay over ten days as measured by the corresponding IM numerical index.

### Instantaneous detection of mechanical damage in pear tissue

A controlled impact test using a 0.6-cm steel ball created invisible bruises in pear tissues. Biospeckle laser imaging successfully detected damaged regions through temporal contrast mapping. The assay was further tested and evaluated on ten different pear samples and similar results on the biospeckle activity were obtained.

**Figure 6:**
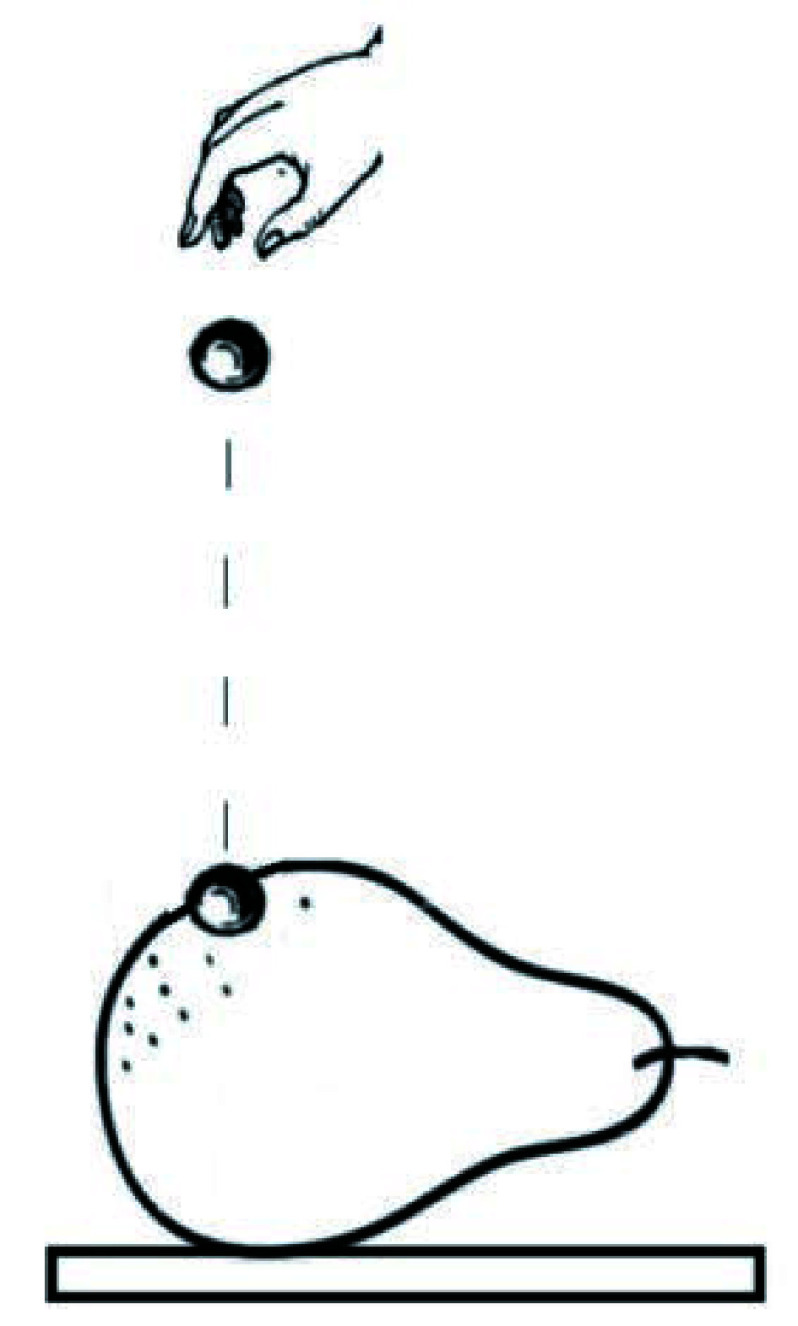
Instant mechanical damage caused by letting metal ball on pear surface

**Figure 7:**
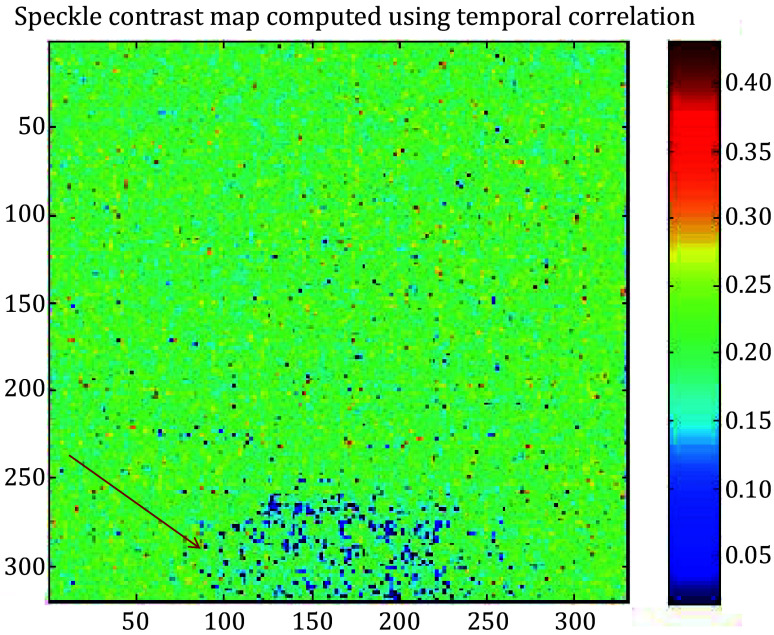
Detection of instant invisible bruise region in a biological sample (pear). The bruised region shows the lowest biospeckle activity compared to the healthy area

Instant mechanical damage to the fruit (pear) tissue was caused by letting a small metal (steel) ball with a diameter of 0.6 cm from a height of 50 cm as shown in Fig. 6. This resulted in instant and invisible mechanical damage in the fruit tissue. Fruit containing the damaged area was then subjected to biospeckle laser imaging. A stack of time-series speckle video sequence data was collected and processed using graphic processing by Matlab R2008a software, according to the speckle contrast evaluation using tLASCA (White *et al*. 2011), which produced visual maps of the damaged area as shown in Fig. 7.

Figure 7 shows the activity map of the fresh fruit surface tissue, which clearly identifies the damaged tissue within the healthy region. It was an instant and invisible mechanical damage introduced into the fruit tissue. Measurements were performed immediately after the impact and the data was processed using an algorithm described by Eq. 5 (shown in the Material and Methods section). Figure 7 shows the processed activity image of the assay. The damaged area displays relatively lower activity shown in blue color in the pseudo-color image. The position of impact is surrounded by the area with high biospeckle activity. Biospeckle processing can thus be an efficient, online, and real-time imaging modality for biological processes related to cells and tissues.

**Figure 8:**
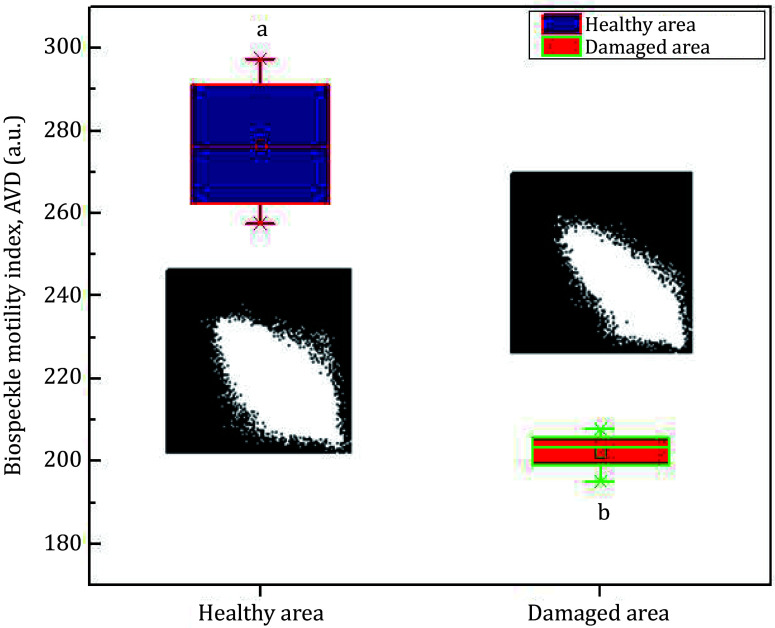
Detection of instant invisible bruise region in a biological sample (pear). The bruised region shows the lowest biospeckle activity compared to the healthy area of the sample. On each box, the line in the center is the median, the small square is the mean, and the edges of the boxes are the 25% and 75%; the whiskers present the most extreme data considering the outliers

A quantitative evaluation of the assay was done using a numerical computation algorithm of AVD as defined by Eq. 7. Figure 8 shows the box plot of the AVD computation both for the healthy region and the damaged area of the fruit. The results shown represent the averaged data on ten different samples. As measured by the AVD index, the damaged region shows relatively lower biospeckle activity than was expected. The values are statistically significant and different (see the indices on the graph, statistical value *a* for the healthy region and *b* for the damaged region). On the other hand, the healthy part of the fruit maintains a higher biospeckle activity.

**Figure 9:**
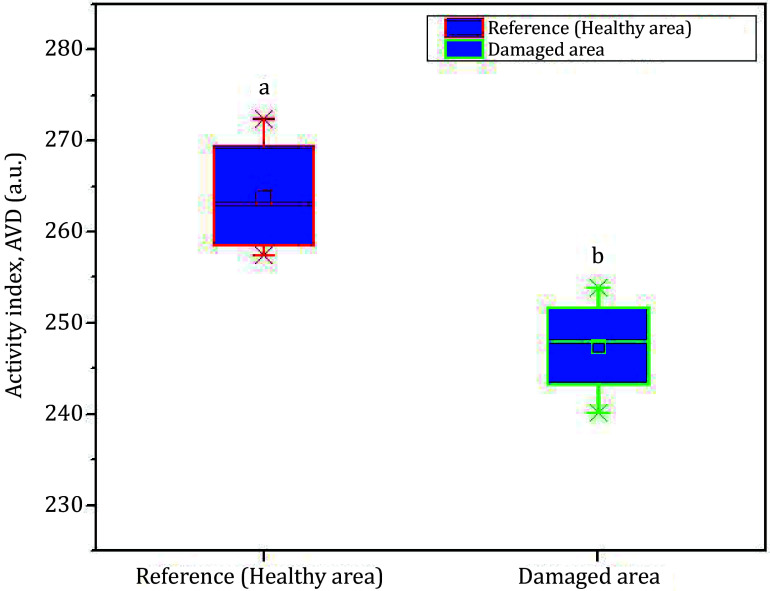
Detection of instant invisible bruise region in a biological sample (apple). The bruised region shows the lowest biospeckle activity compared to the healthy area of the sample. Where on each box, the line in the center is the median, the small square is the mean, and the edges of the boxes are the 25% and 75%; the whiskers present the most extreme data considering the outliers

A similar study was performed on an apple and a statistically significant differences between the activity values of the two areas, the damaged one and the healthy one. Figure 9 shows a box plot of the corresponding AVD values of either of the areas of the fruit. The data shown is averaged over ten different samples of apples. The graph again shows a high biospeckle activity in the healthy region compared to the damaged area of the fruit.

### Activity evaluation in orange fruit scar tissue

Analysis of the peduncle insertion region revealed low activity due to its scar-like nature. To determine the activity of the scar zone in fresh green orange, where the peduncle was inserted, a second experiment was conducted. Fresh, healthy, and green orange fruits were sacrificed using the biospeckle laser method for experimental investigation. As shown in Fig. 10.1, points A and B represent diametrically opposed locations in the equatorial zone, while points C and D represent the fruit's apex area and the insertion of the peduncle, respectively. This investigation attempted to assess the biospeckle activity of the peduncle insertion region, which exhibits low activity because the peduncle insertion site acts as a scar (Rabelo *et al*. 2005).

**Figure 10:**
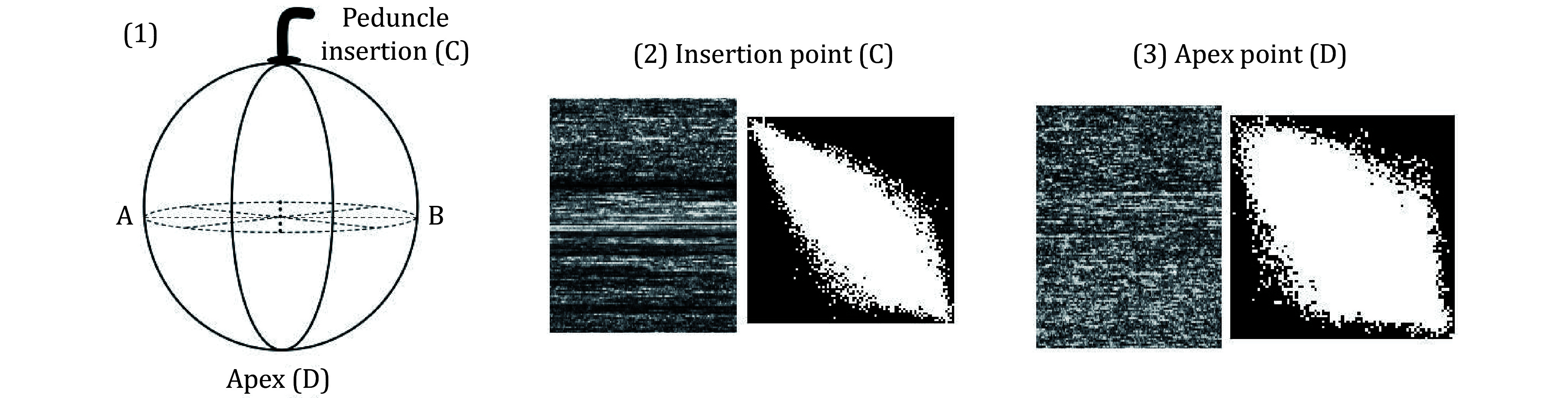
THSP and co-occurrence matrices for the two regions of an orange at (2) insertion point (C) and (3) apex point (D)

Temporal information can be extracted by constructing a 2D map called THSP, the vertical direction of which shows the time evolution of the phenomena. A specimen with high activity had a THSP image that resembled a general speckle pattern, because the corresponding biospeckle pattern changed quickly.

However, in samples with little activity, the THSP pattern appeared as parallel vertical bars. It is possible to ascertain the evolution of the activity by generating a COM matrix and computing the numerical index, IM. Activity points were found on the major diagonal of the COM matrix. The sample that showed high activity saw dots on the COM matrix becoming more scattered.

### Oxidation activity in cut apple surfaces

Comparative THSP and COM matrix analyses demonstrated increased biospeckle activity in freshly cut apple surfaces due to oxidative enzyme reactions.

The experiment was performed to evaluate the activity of fresh and cut apple surfaces using biospeckle analysis. The cutting of an apple causes oxidation, a chemical reaction that occurs when oxygen enters the plant tissue. Polyphenol oxidase (PPO) enzyme is found in apples. Melanin is a dark pigment created when this enzyme combines with oxygen to transform colorless substances.

**Figure 11:**
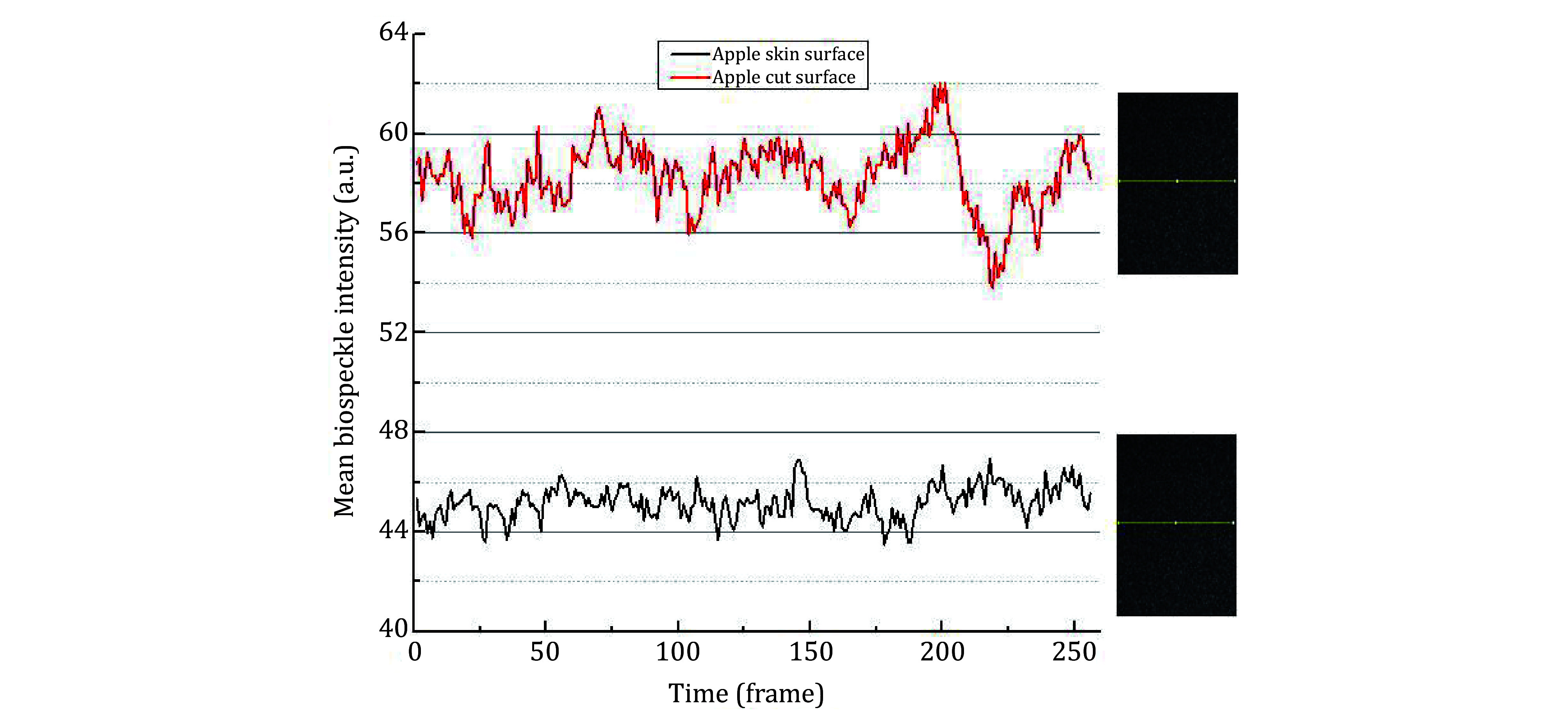
Mean activity of fresh skin and cut surfaces of an apple

Figure 11 shows the time evolution of dynamic speckle intensity along a horizontal line (yellow line in the corresponding speckle image). Because of the oxidation on the cut surface, the assay relatively shows higher activity and this has caused the biospeckle intensity value comparatively high and produced more dynamic fluctuations as shown in Fig. 11.

**Figure 12:**
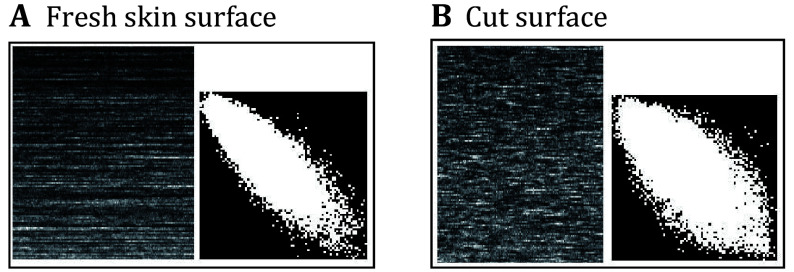
Biospeckle activity evolution of a fresh and cut apple surface. The corresponding THSP and COM matrices on the fresh apple surface (**A**) and its cut surface (**B**)

Figure 12 shows THSP and COM matrices of fresh skin surface and cut surface. These matrices can be used to characterize the activity of the assay. From Fig. 12, one can observe that the fresh-cut surface shows relatively more activity than the fresh skin area of the fruit. The THSP image, in the case of the skin surface, displayed an elongated shape, and its corresponding COM matrix predicted its values near its diagonal. However, this value is more scattered about the diagonal as the THSP matrix shows randomness in its pixel values, similar to a speckle image.

**Figure 13:**
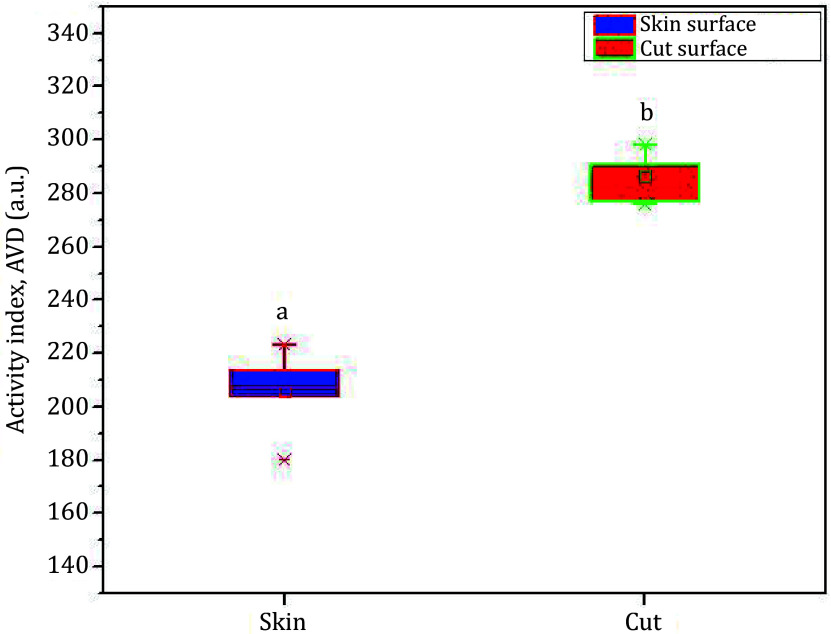
Evaluation of biospeckle activity in fresh skin surface and that in the cut surface of an apple. The fresh-cut region exhibits higher biospeckle activity. On each box, the line in the center is the median, the small square is the mean, and the edges of the boxes are the 25% and 75%; the whiskers present the most extreme data considering the outliers

Figure 13 presents the biospeckle activity index, showing the AVD values for both surfaces of the fruit — the fresh skin surface and the freshly cut surface. Due to oxidation on the cut surface, the assay exhibits a relatively higher activity, leading to increased biospeckle activity. The results shown represent averaged data on ten different samples. The values were found statistically significant and different. The statistical indices (*a, b*) in the graph demonstrate the validity of the results.

## CONCLUSIONS

We measured the statistical characteristics of the biospeckle patterns of different biological specimens. In order to track the senescence of vegetal tissue, we found that the WPF method worked well for creating maps of the vascularization of leaf tissues. This technique relies on the presence of fluid flow to create detailed maps of tissue microcirculation. The method of temporal contrast evaluation produced a significant spectral activity map, which allowed for the detection of both instant and invisible bruised tissue. A quantitative evaluation of the AVD index served as a biological signature for the sample. This allowed us to differentiate between the activity values at two different points on a fruit, as well as between fresh and cut fruit surfaces. These findings validate biospeckle imaging as a real-time, non-invasive diagnostic tool for biological applications.

## DISCUSSIONS

While the biospeckle laser imaging system provided valuable insights, some limitations remain. The technique is highly sensitive to external vibrations, requiring a stable environment for accurate measurements. Additionally, variations in sample heterogeneity may introduce inconsistencies in biospeckle activity maps. Future improvements could involve enhanced image processing algorithms to minimize noise and improve signal interpretation. Further research could also explore real-time monitoring applications in clinical diagnostics and agriculture, potentially integrating AI-based analysis for automated evaluation.

## MATERIALS AND METHODS

### Experimental setup

Two fundamental setups are typically used for biospeckle attainment. In Fraunhoffer's plane, the first configuration is typically employed to evaluate biospeckle because the free-space geometry forms a grainy pattern. Here, surface evaluation was performed locally. The phenomena can be tracked in the image plane in the second configuration, making it possible to examine the spatial distribution of activity throughout the specimen.

Figure 1 shows an experimental setup for biospeckle imaging. A backward scattering configuration was used to illuminate the sample. A 3-mW He−Ne laser operating at 632.8 nm was used to illuminate the leaf surfaces. The sample was covered with a spatially filtered and enlarged laser beam. The photos were taken using a CCD camera (Basler, Germany) with a resolution of 1294 × 964 pixels, a frame rate of 32 frames per second, and a pixel size of 3.75 μm × 3.75 μm. To achieve low specular reflection of light and maximize contrast in the photographs, the angle at which the laser beam was focused on the CCD camera was modified.

### Biospeckle data processing

Time-series light intensity patterns were acquired using biospeckle laser imaging. The temporal and spatial variations in pixel intensities provided insights into cellular activity and microflow.

#### Microcirculation mapping in leaf tissues

Two primary techniques were employed: the Fujii method and the TD method. Each generated grayscale images, where high gray levels indicated higher biological activity. For a sequence of *n* integer values *s* = [*x*_0_, *x*_1_, …, *x*_*n*−1_], the Fujii function *F*_*p*_(*s*), is defined by Eq. 1 (Ansari and Nirala 2015):

1 $ F_p\left(s\right)=\sum\limits_{i\ =\ 0}^{n-1}\frac{\left|x_i-x_{i+1}\right|}{x_i+x_{i+1}}. $

The TD algorithm calculates the sum of absolute differences of consecutive images separated by a time interval, and it generates an activity map using the algorithm defined by Eq. 2 (Retheesh *et al*. 2018):

2 $ TD\left(s\right)=\sum\limits_{i\ =\ 0}^{n-1}\left|x_i-x_{i+1}\right|. $

An equation that is generalized and capable of valorizing locations around any gray level of interest should be the optimal approach. Our suggestion is weighted parameterized Fujii, a parameterized variant of the original Fujii method, which is defined as an alternative. This method enables the user to adjust its parameters based on the objects of interest present in the images (Ansari and Nirala 2015).

A refined WPF method was introduced to enhance sensitivity to subtle gray-level variations. The *F*_wp_ (*s*, *g*_*r*_) for a sequence of *n* integer values *s* = [*x*_0_, *x*_1_, …, *x*_*n*-1_], weighted by values around a reference gray level *g*_*r*_, is defined by Eq. 3 (Ansari and Nirala 2015):

3 \begin{equation*}\begin{split}
F_{wp}\left(s,g_r\right)= & \sum\limits_{i\ =\ 0}^{n-1}\frac{\left|x_i-x_{i+1}\right|}{x_i+x_{i+1}}(255-\left|g_r-x_i\right| \\ &+255-\left|g_r-x_{i+1}\right|)=\sum\limits_{i\ =\ 0}^{n-1}\frac{F_p\left(s,g_r\right)}{\left|x_i+x_{i+1}\right|},
\end{split}\end{equation*}

where ${F_p}( {s,{g_r}} )  =  | {{x_i}  -  {x_{i + 1}}} |( 255  -  | {{g_r}  -  {x_i}} |  +  255  -  | {g_r}  -  {x_{i + 1}} | )$.

The weighting term, represented by the summation of the two subsequent images, emphasizes small changes, which provide a clearer image than the parameterized Fujii and alternative Fujii images (Ansari and Nirala 2015).

#### Speckle contrast evaluation using temporal correlation (tLASCA)

The movement of scatterers creates a blurring of the speckle pattern in the obtained image when a time-varying speckle pattern is photographed with a finite-exposure-time CCD camera. The speckle contrast value, *K*, is given by Ansari *et al*. and Briers (Ansari *et al*. 2016; Briers 2001):

4 $ K = \frac{\sigma }{{\left\langle I \right\rangle }}, $

where *σ* is the standard deviation and 〈*I*〉 is the mean intensity of a pixel group.

The temporal speckle contrast (tLASCA) method refines this calculation for dynamic analysis (Briers 2001; White *et al*. 2011). This method differentiates slow- and fast-moving speckles, aiding in microcirculation assessment.

For each frame, the contrast value *K*_*t*_ of pixel (*i, j*) of a particular frame is computed by Eq. 5 (White *et al*. 2011) :

5 $ K_t\left(i,j\right)=\sum\limits_{r\ =\ i-1}^{i\ +\ 1}\sum\limits_{c\ =\ j-1}^{j\ +\ 1}\frac{\delta_{r,c,t}}{\left\langle I_{r,c,t}\right\rangle}, $

where *δ*_*r*,*c*,*t*_ is the standard deviation of all pixels at *(r, c)* and 〈*I*_*r*,*c*,*t*_〉 is the mean intensity of all pixels at *(r, c)* in *n* frames along the temporal dimension. Usually, the difference between slow and rapidly moving speckles, which match the mean and standard deviation, respectively, is identified using this method.

#### Quantitative activity index evaluation

Two intensity-based numerical methods can be used to analyze the time-varying speckle pattern activity: calculating the THSP image or utilizing the correlation coefficient to compare the recorded speckle images with the first image in the series.

The first technique for tracking the temporal fluctuation of speckle pictures over time is the temporal history of speckle pattern (THSP) (Ansari and Nirala 2016; Rabelo *et al*. 2005). It is produced by first taking N continuous speckle images and then selecting the center column from each image. The THSP, a new image, was created by registering *N*-selected columns.

A shift in intensity in the horizontal direction is indicative of sample activity in THSP. The THSP had an extended form resembling parallel bars when the sample exhibited low activity. When the phenomenon is active, the THSP appears to display a typical speckle appearance.

The gray-level COM, can be used to examine THSP images, which is described as follows (Ansari and Nirala 2014).

The values represent the number *N* of instances of an intensity value *i* that is immediately followed by another intensity value *j*. Regarding the spatial instance, regions with homogenous values are associated with their primary diagonal, while nonzero elements located at a distance from it signify high-contrast events. The intensity values fluctuate over time as the sample demonstrates activity, the number *N* outside the diagonal rises, and the matrix takes on the appearance of a cloud. The activity of the assay can be quantified by the degree of the scattering of the number *N* about the diagonal. One of the ways to define the activity is to measure the IM of the COM matrix as described by the following equation:

6 $ IM = \sum\limits_{ij} {CO{M_{ij}}{{\left( {i - j} \right)}^2}} . $

In the current study, we evaluated the assay numerically in relation to its behavior in time using the absolute value of difference (AVD) (Ansari *et al*. 2021; González-Peña *et al*. 2014; Ansari 2024). The activity of the assay can be quantified by using Eq. 7:

7 $ AVD = \sum\limits_{ij} {CO{M_{ij}}\left| {i - j} \right|} , $

where the dimensions of the COM matrix are defined by variables *i* and *j*, and COM is associated with the THSP matrix (Ansari *et al*. 2021; González-Peña *et al*. 2014; Ansari 2024).
